# Rat model of an autologous cancellous bone graft

**DOI:** 10.1038/s41598-021-97573-0

**Published:** 2021-09-09

**Authors:** Tomo Hamada, Hidenori Matsubara, Toshifumi Hikichi, Kanu Shimokawa, Hiroyuki Tsuchiya

**Affiliations:** Department of Orthopedic Surgery, Graduate School of Medical Science, Kanazawa University, 13-1 Takara-machi, Kanazawa, 920-8641 Japan

**Keywords:** Biological models, Experimental models of disease, Musculoskeletal system

## Abstract

Autologous cancellous bone (ACB) grafting is the “gold standard” treatment for delayed bone union. However, small animal models for such grafts are lacking. Here, we developed an ACB graft rat model. Anatomical information regarding the iliac structure was recorded from five rat cadavers (10 ilia). Additionally, 5 and 25 rats were used as controls and ACB graft models, respectively. A defect was created in rat femurs and filled with ACB. Post-graft neo-osteogenic potential was assessed by radiographic evaluation and histological analysis. Iliac bone harvesting yielded the maximum amount of cancellous bone with minimal invasiveness, considering the position of parailiac nerves and vessels. The mean volume of cancellous bone per rat separated from the cortical bone was 73.8 ± 5.5 mm^3^. Bone union was evident in all ACB graft groups at 8 weeks, and new bone volume significantly increased every 2 weeks (P < 0.001). Histological analysis demonstrated the ability of ACB grafts to act as a scaffold and promote bone union in the defect. In conclusion, we established a stable rat model of ACB grafts by harvesting the iliac bone. This model can aid in investigating ACB grafts and development of novel therapies for bone injury.

## Introduction

Autologous cancellous bone (ACB) grafts are considered the “gold standard” treatment for post-traumatic bone conditions, including fracture, delayed union, and nonunion^1^. Cancellous autografts contain osteoblasts, mesenchymal stem cells, bone morphogenetic protein, and growth factors. The cancellous matrix serves as an excellent scaffold for vascular ingrowth and osteoblastic cell infiltration owing to its osteogenic, osteoinductive, and osteoconductive properties^2–6^, making it a potential candidate for the treatment of acute and reconstructive traumas. However, limitations, including donor-site morbidity and graft availability, render autografts suboptimal for some patients. Nevertheless, advances in allografts and artificial bone grafts have made them viable alternatives owing to their convenient application, abundance, and absence of procurement-related patient morbidity. Although allografts or artificial bone grafts have bone union properties^7–9^, autologous bones remain the “gold standard” in orthopedic surgery due to ethical reasons and because the bone union property of the autologous cancellous bone is superior to that of allografts or artificial bone grafts^10–12^.

Studies on bone grafts in various rat models have employed the strategy of grafting natural bovine cancellous bone particles into the alveolar ridge^13^, implanting heat-treated porcine cancellous bone particles into the calvarial defects^14^, and implanting freeze-dried cancellous bone of the tibia of other rats^15,16^. An advantage of rat models with an allograft is that they do not require invasive bone collection. However, as freeze-dried or heat-treated bone grafts have lower osteogenic, osteoconductive, and osteoinductive potentials than bone autografts, bone grafts implanted in these models differ from clinically used autologous bone grafts. Furthermore, additional rats are required for bone collection, making the process cost-intensive. Although various medium- and large-sized animal ACB models have been established^17–20^, effective small animal models, capable of recapitulating the clinical conditions of ACB grafts, have not been developed. Small animal models can provide important insights into disease pathogenesis and the underlying molecular pathways, thereby facilitating the development of novel therapeutic agents and strategies. Therefore, it is necessary to establish new animal models for orthopedic research.

Accordingly, in this study, we aimed to determine whether collecting rat ilium is a safe procedure and to determine the volume (and whether it is sufficient) of cancellous bone that can be obtained from the ilium. Moreover, we analyzed the ability of the collected cancellous bone to promote bone union.

## Methods

### Animal experiments

All animal experiments complied with the ARRIVE guidelines, and the Institutional Ethics Committee at Kanazawa University Advanced Science Research Center approved all experimental protocols of this study (approval no.: AP-184005). All methods were performed in accordance with the relevant guidelines and regulations. Thirty-five healthy male Sprague–Dawley rats, aged 12 weeks and weighing 374.8 g (range 350–400 g), were obtained from Charles River Laboratories, Inc. (Wilmington, MA, USA). Of note, these rats were not previously exposed to any specific drug. The rats were randomly allocated to three groups, namely, group A (n = 5)—subjected to iliac ACB harvesting—and groups B (n = 25) and C (n = 5) (described below). The rats were individually housed in cages under specific pathogen-free conditions, with a 12-h light/dark cycle and free access to food and water and were acclimatized for 1 week in the laboratory before the experiments. The rats were anesthetized with an intraperitoneal injection of medetomidine (0.15 mg/kg), midazolam (2 mg/kg), and butorphanol (2.5 mg/kg) in all experiments. Analgesia was induced via subcutaneous administration of buprenorphine (0.01 mg/kg) before and immediately after surgery. The rats were euthanized via intraperitoneal injection of secobarbital (450 mg/kg). Two rats in group B died during the experiment and were thus excluded from the analyses.

### Anatomy of ACB for iliac bone harvesting

Bilateral ilia from five freshly frozen male Sprague–Dawley rat cadavers from group A were dissected to determine the formation ilium and organization of parailiac nerves and vessels. We measured the distance from the top of the ilium to the iliac crests to determine the safest osteotomy line (n = 10). We then determined the maximum volume of stable cancellous bone by measuring the total volume of cancellous bone in the harvested iliac crests by computerized tomography (CT). Thereafter, the cancellous bone was separated from the cortical bone in all iliac crests, and its total volume was measured in the same way. The combined cancellous bone volume obtained from the left and right iliac crests represented the total volume collected from one rat. The cancellous bone was then embedded in paraffin for hematoxylin and eosin (HE) staining and observed by optical microscopy.

### ACB graft model

**Figure 1:**
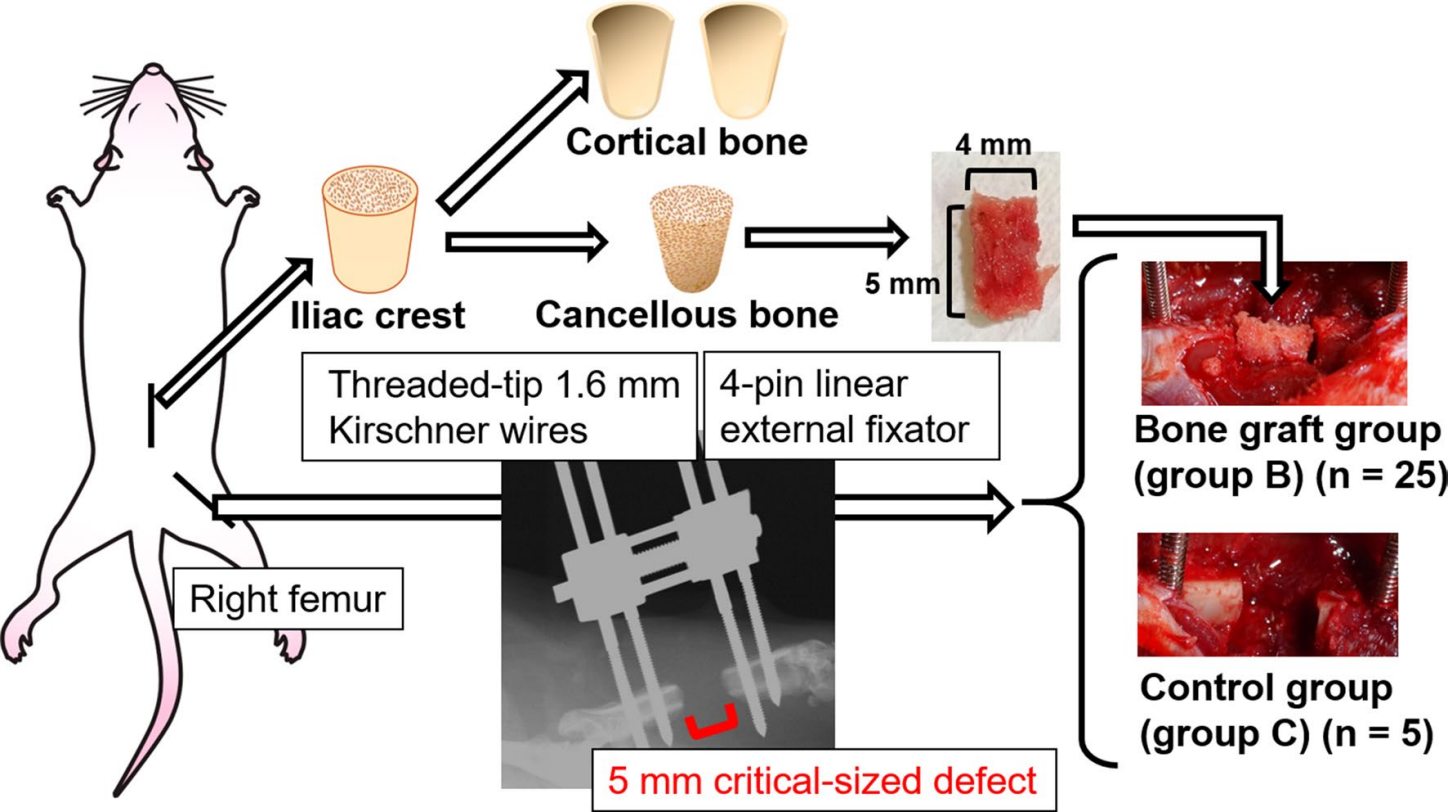
Schematic representation of the experimental procedures for transplanting autologous cancellous bone into a femoral bone defect in rats.

Figure 1 shows an overview of the experimental design. The femoral bone defect model was adapted from previous studies that showed nonunion without implant^21,22^. Each animal was placed in a lateral position on the operating table. A lateral longitudinal skin incision was created over the right femur, followed by an incision and subsequent separation of the quadriceps femoris and hamstrings. After predrilling with a Kirschner wire (1.4 mm diameter), an external fixator (Meira, Nagoya, Japan) was fixed with four self-tapping pins (1.6 mm diameter; Japan Medicalnext, Osaka, Japan) in the femur. Two osteotomies were performed between the second and third pins using a manual saw under irrigation with physiological saline to create a 5-mm segmental defect. The femoral bone defect in group B was filled with the cancellous bone harvested from the bilateral ilia. The femoral bone defect in group C was left without an implant. Muscle, subcutaneous tissue, and skin were closed with simple interrupted sutures, and the rats were returned to their cages without immobilization. The rats in group B (n = 5 per time point) were euthanized 2, 4, 6, and 8 weeks post surgery. To assess bone formation, digital images were obtained, and histological analyses were performed on rats for which CT images were obtained immediately before euthanasia. Furthermore, to determine the progression of bone formation, the right femurs of five rats in groups B and C were immediately assessed by X-ray CT, as well as every 2 weeks for 8 weeks post surgery.

### ACB harvesting

**Figure 2:**
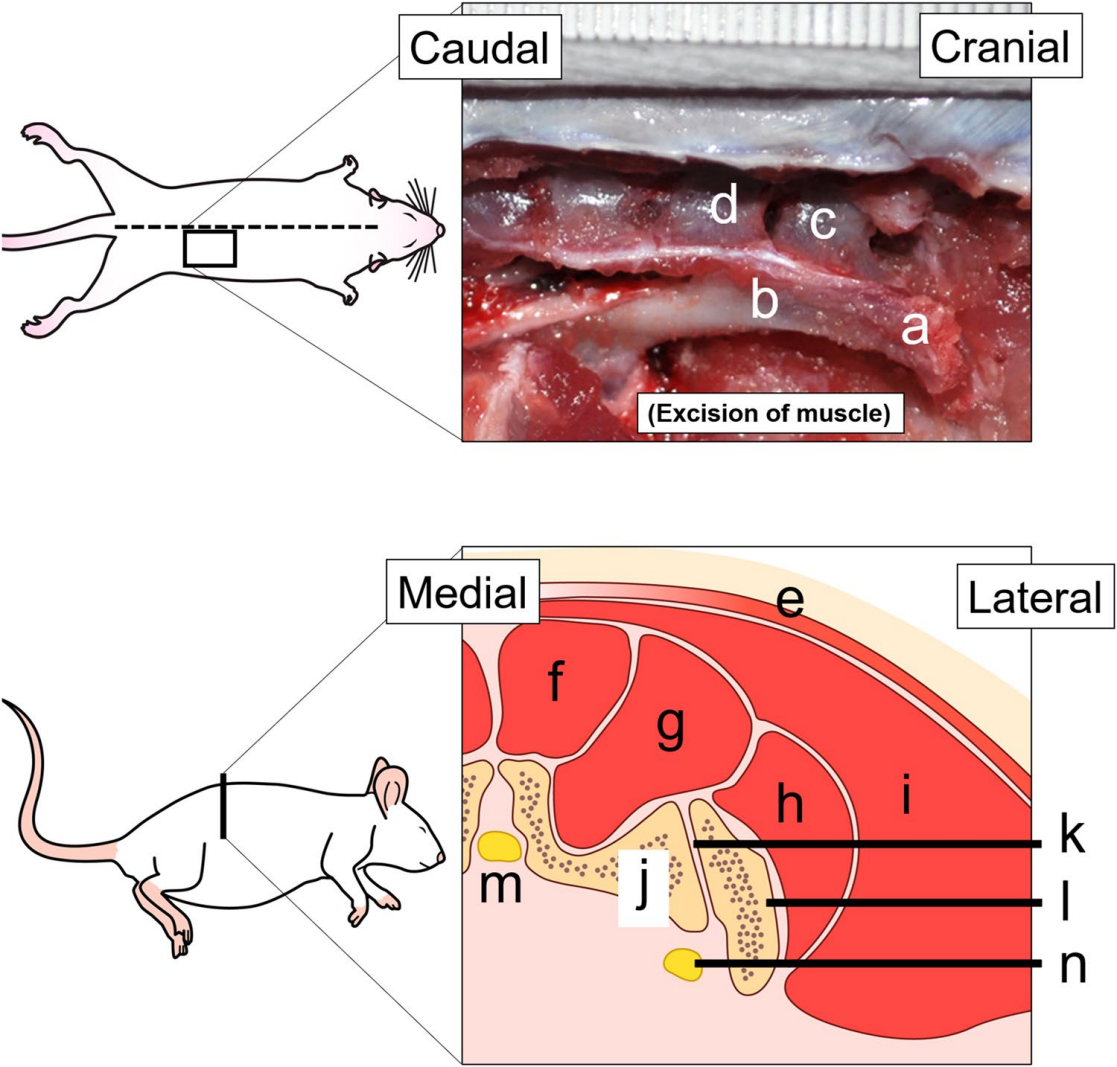
Dissection of the iliac and parailiac regions. The upper figure shows the iliac and parailiac regions from above. (**a**) Iliac crest. (**b**) Ilium. (**c**) First transverse process of the cranial sacrum. (**d**) Second transverse process of the cranial sacrum. The lower figure shows an axial section of the iliac and parailiac regions. (**e**) Cutaneous muscle from the trunk. (**f**) *Sacrococcygeus dorsalis* medialis muscle. (**g**) *Sacrococcygeus dorsalis* lateralis muscle. (**h**) Gluteus minimus muscle. (**i**) *Gluteus maximus* muscle. (**j**) Transverse process of the sacrum. (**k**) Sacroiliac joint. (**l**) Ilium. (**m**) Sacral canal. (**n**) Ischiatic nerve.

**Figure 3:**
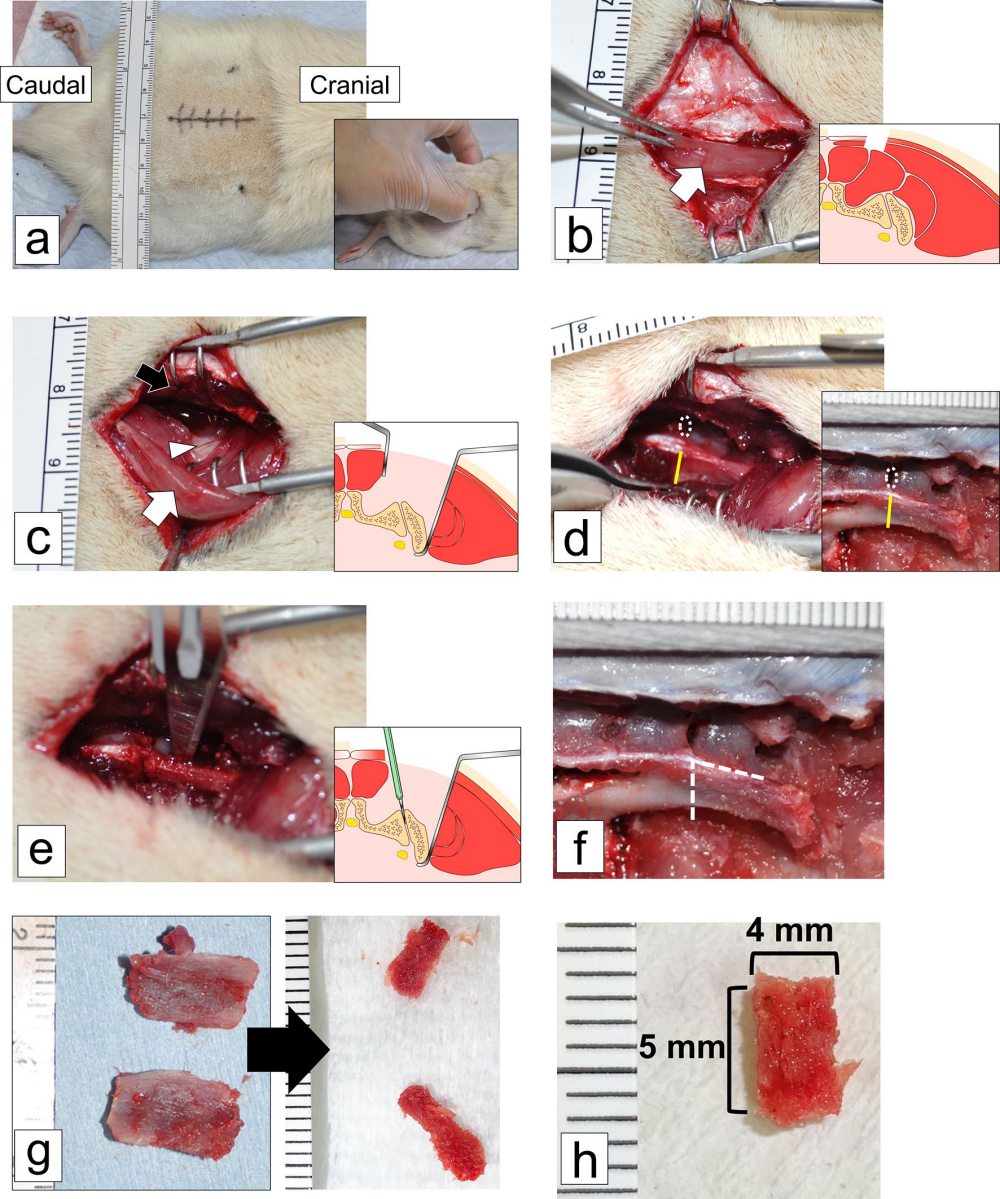
Surgical harvesting of autologous cancellous bone. (**a**) Bilateral iliac crests located on the skin surface; a single ~ 4 cm vertical incision was made in the midline dorsal region, around the highest point of each crest. (**b**) The cutaneous muscle was separated from the trunk and gluteus maximus, laterally (5 mm) to the dorsal midline. White arrow, sacrococcygeal dorsalis lateralis muscle. (**c**) The sacrococcygeal dorsalis and dorsalis lateralis muscles were separated to access the iliac crest. White arrow, sacrococcygeal dorsalis lateralis muscle; black arrow, sacrococcygeal dorsalis medialis muscle; white arrowhead, iliac crest. (**d**) Transversal osteotomy of the ilium between the lateral first and second cranial parts of the sacrum. White dotted circle, hole between the first and second transverse processes of the cranial sacrum; yellow line, osteotomy line. (**e**) The sacroiliac joint was separated between the lateral part of the sacrum and the ilium using a scalpel. (**f**) Both iliac crests were harvested from each rat. White dotted line, osteotomy line. (**g**) Cancellous bone was separated from the cortical bone in the iliac crests. (**h**) The morselized cancellous bone was placed in cylindrical molds (diameter, 4 mm) and compressed into 5 mm blocks. Scale bar: 1 mm.

We examined the anatomy of rats by referring to a previous study^23^ (Fig. 2) and harvested ACB as follows: (i) identified bilateral iliac crests on the skin surface and made a single ~ 4 cm vertical incision along the midline dorsal region around the highest point of each crest (Fig. 3a); (ii) separated the cutaneous muscle from the trunk and the gluteus maximus muscle 5 mm lateral to the dorsal midline (Fig. 3b); (iii) separated sacrococcygeal dorsalis medialis and lateralis muscles to access the iliac crest (Fig. 3c); (iv) performed transversal osteotomy of the ilium between the first and second transverse processes of the cranial sacrum (Fig. 3d); (v) separated the sacroiliac joint between the first transverse process of the cranial sacrum and the ilium, using a scalpel (Fig. 3e); (vi) harvested both iliac crests from each rat (Fig. 3f); (vii) closed the cutaneous muscles of the trunk and gluteus maximus muscle with simple interrupted sutures and stitched the skin; (viii) separated the cancellous bone from the cortical bone in iliac crests using standard pointed and circular scalpels (Fig. 3g) and morselized it using a sharp scalpel; and (ix) placed the morselized cancellous bone in cylindrical molds (diameter, 4 mm) and compressed into 5 mm blocks (Fig. 3h).

### Radiographic evaluation

All femurs were evaluated by X-ray micro-CT on a LaTheta LCT-200 CT system (Hitachi-Aloka, Tokyo, Japan)^24^, and DICOM viewer software, Onis version 2.5 (DigitalCore Co., Ltd., Tokyo, Japan) was used to quantify DICOM data. The femoral axis was set using coronal images, and bone formation was evaluated only in the 5 mm (200 slices) central defect region to ensure that no preexisting cortical bone was included in the analyses. The CT values were calibrated to those for water (CTw = 0) and air (CTa =  − 1000), and areas with CT values above þ1000 were extracted as new bone volumes.

### Histological analysis of tissues

The femurs harvested at 2, 4, 6, and 8 weeks post surgery, with the surrounding soft tissue and external fixator attached (n = 5 per time point), were fixed in 10% neutralized formalin and dehydrated using an ethanol gradient (70%, 80%, 90%, and 100%). The fixed specimens were decalcified in 10% formic sodium citrate, and the external fixator was removed. The specimens were embedded in paraffin, sectioned in the sagittal plane, stained with HE and Safranin O, and assessed using a Biorevo BZ-9000 optical microscope (Keyence Co., Osaka, Japan).

### Statistical analysis

Power analysis using G*Power showed that to detect a 25% difference in bone growth with statistical significance (α = 0.05; power = 0.8) at the known level of variance, calculated from mean and standard deviation published by pilot studies, we required five rats in both control and experimental test groups. Data were subjected to Shapiro–Wilk normality test and statistical analyses using Statistical Package for Social Sciences version 23.0 (IBM Corp., Armonk, NY, USA). Results are presented as mean ± standard deviation. The data were normally distributed; thus, paired comparisons of bone growth rates at various time points were performed using the ANOVA. Differences were considered significant at *P* < 0.05.

## Results

### Anatomy of an ACB for iliac bone harvesting

**Figure 4:**
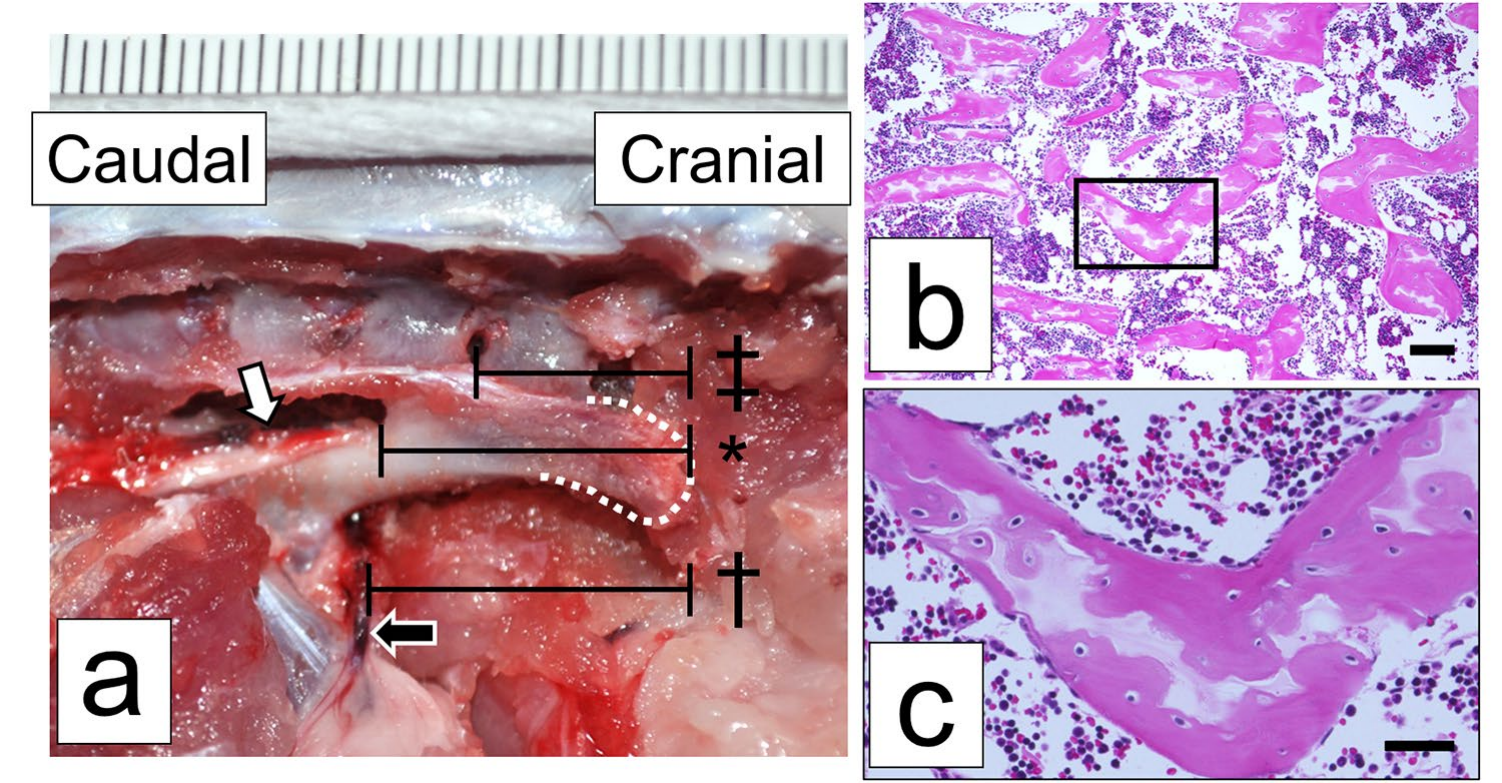
Anatomy associated with harvesting of autologous cancellous bone from the ilium. (**a**) The iliac bone and organization of the parailiac region. White dotted line, top of the iliac crest; white arrow, ischiatic nerve; black arrow, ischiatic vein. Distance from the top of the iliac crest to the (*) intersection of the ischiatic nerve, ^†^intersection of an ischiatic vein, and ^‡^hole between the lateral and first and second cranial parts of the sacrum. Scale bar: 1 mm. (**b**) Hematoxylin and eosin-stained sections of the harvested bone confirmed to be cancellous. Scale bar: 100 μm. (**c**) Higher magnification of the inset in (**b**). Scale bar: 200 μm.

Similar to humans, rats have iliac crests that are not surrounded by important vessels or nerves (Fig. 4a). The ischiatic nerve and vein cross the pelvis at the caudal end of the iliac crest and enter the pelvis (Fig. 4a). The mean distance from the top of the iliac crest to the intersection of the ischiatic nerve or vein with the pelvis was 15.5 ± 0.55 and 18.8 ± 0.94 mm, respectively (Fig. 4a). The landmark for safe osteotomy was the hole between the first and second transverse processes of the cranial sacrum. This hole was easily spotted while harvesting the iliac bone crest. The mean distance from the top of the iliac crest to this hole was 11.4 ± 0.89 mm (Fig. 4a). We set the safest osteotomy line at the level of the hole most cranial to the transverse process of the sacrum. The radiographic evaluation showed that the mean cancellous bone volume in the iliac crest was 132.2 ± 6.3 mm^3^, whereas after separation from the cortical bone, it was 73.8 ± 5.5 mm^3^. Histological findings confirmed that only the cancellous bone with cell nuclei remained after separation (Fig. 4b,c).

### Radiographic evaluation of model ACB grafts

**Figure 5:**
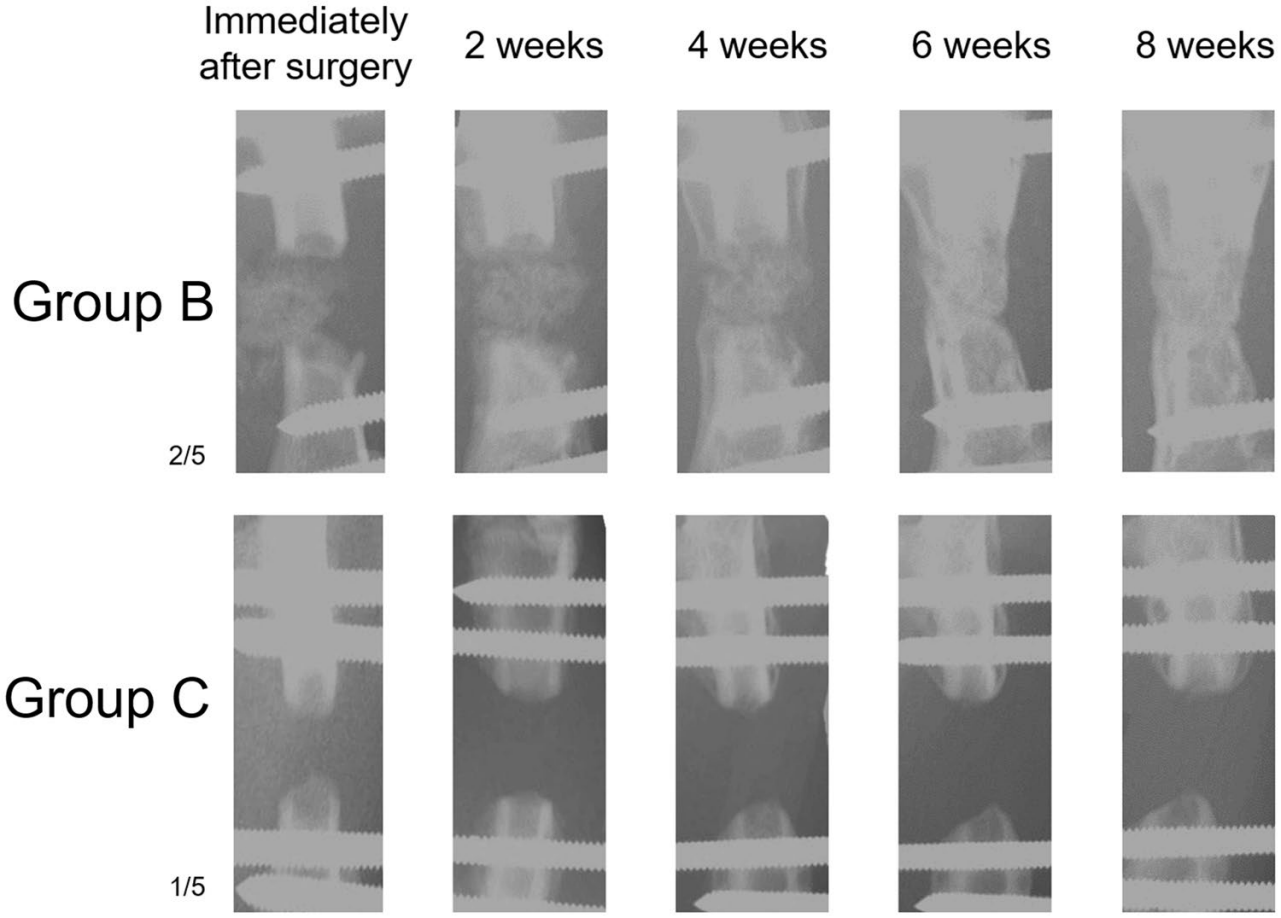
X-Ray computed tomography micrographs of the model rats at sequential time points. An autologous cancellous bone graft in group B and control (group C) over time.

**Figure 6:**
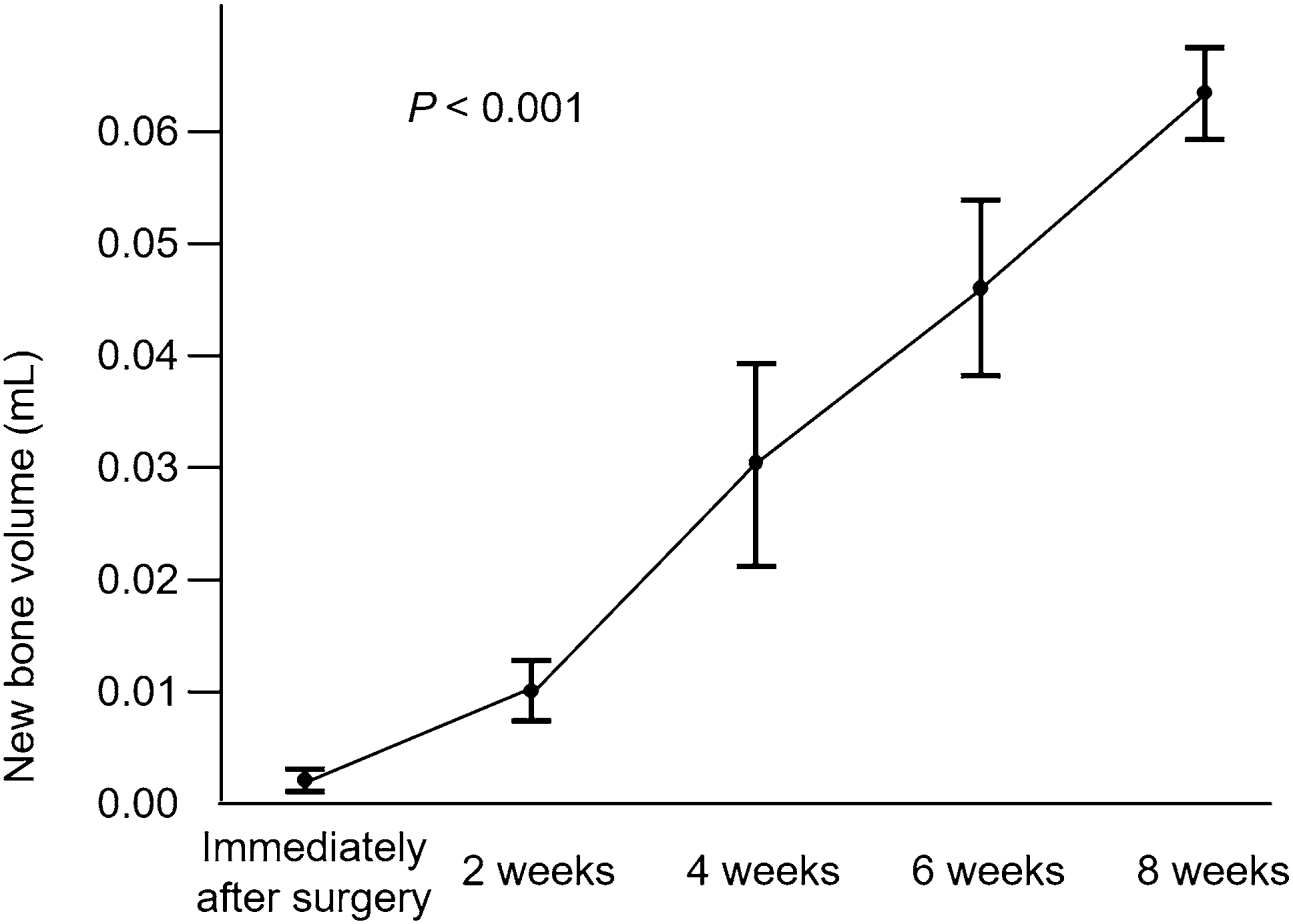
New bone volume in rats from group B (with bone defects). New bone volume was determined as the volume of bone with a computerized tomography value above þ1000 (mL). Data are presented as mean ± standard error of the mean (n = 5). Statistical significance was assessed using the ANOVA.

Figure 5 shows X-ray CT images of representative rats in groups B and C at consecutive time points. Changes suggestive of neo-osteogenesis were confirmed at 2 weeks and were more prominent at 8 weeks post grafting in group B. The increase in new bone volume, measured by X-ray CT, was gradual and significant in group B (*P* < 0.001; Fig. 6). Bone union was evident in the ACB graft groups at 8 weeks, whereas bone did not fuse in the control rats.

**Figure 7:**
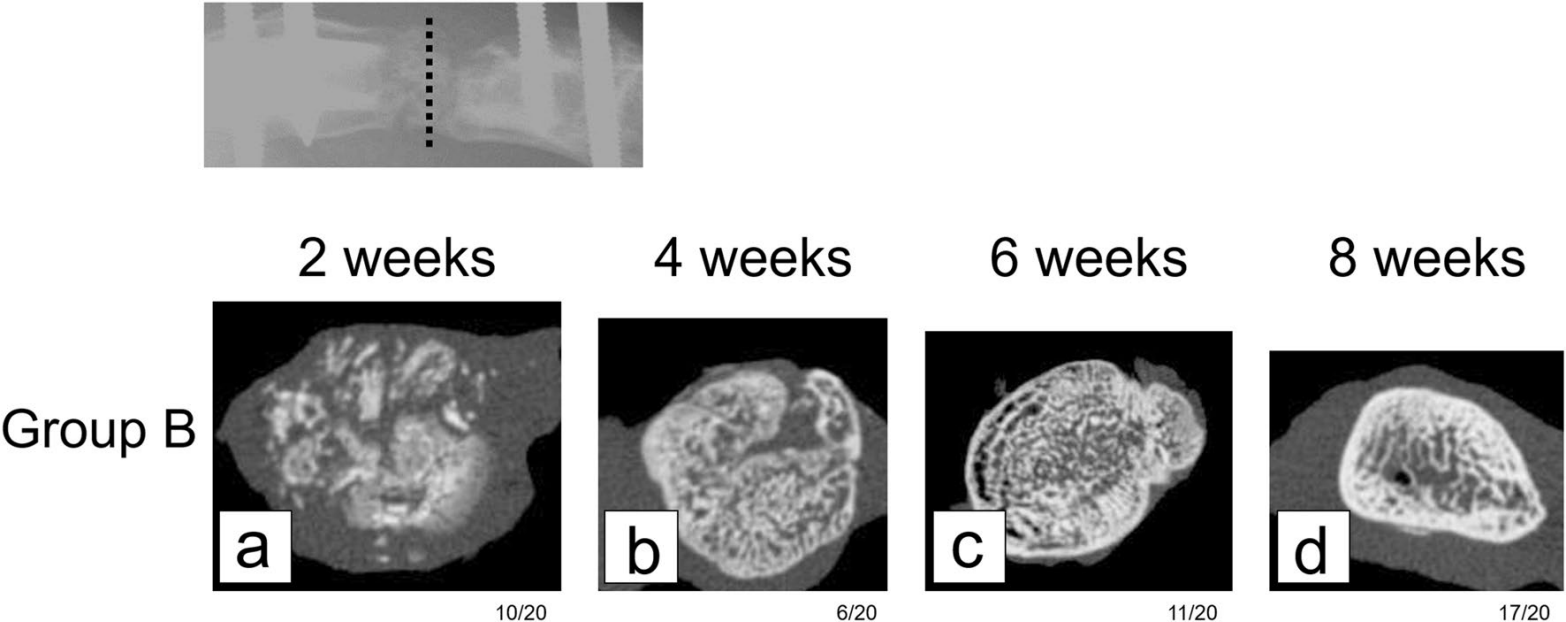
Axial micro-computerized tomography view of the center of the bone defect in model rats euthanized at various time points after surgery (group B). (**a**) Central defect region showed that the bone graft structure at 2 weeks post surgery was only trabecular. (**b**) At 4 weeks, the trabecular structure in the outer periphery of the bone defect was completely replaced by the cortical bone. (**c**) By 6 weeks, the cortical bone had thickened. (**d**) By 8 weeks, new bone appeared slimmer and more compact.

X-Ray micro-CT images acquired for rats in group B showed that the bone graft structure at 2 weeks after surgery was only trabecular (Fig. 7a). At 4 weeks, the trabecular structure in the outer periphery of the bone defect was completely replaced by the cortical bone, indicating that the defect was securely bridged (Fig. 7b). By 6 and 8 weeks, the cortical bone had thickened (Fig. 7c,d). By 8 weeks, the earlier rounded and plump new bone appeared slimmer and more compact.

### Histological analysis of ACB grafts

**Figure 8:**
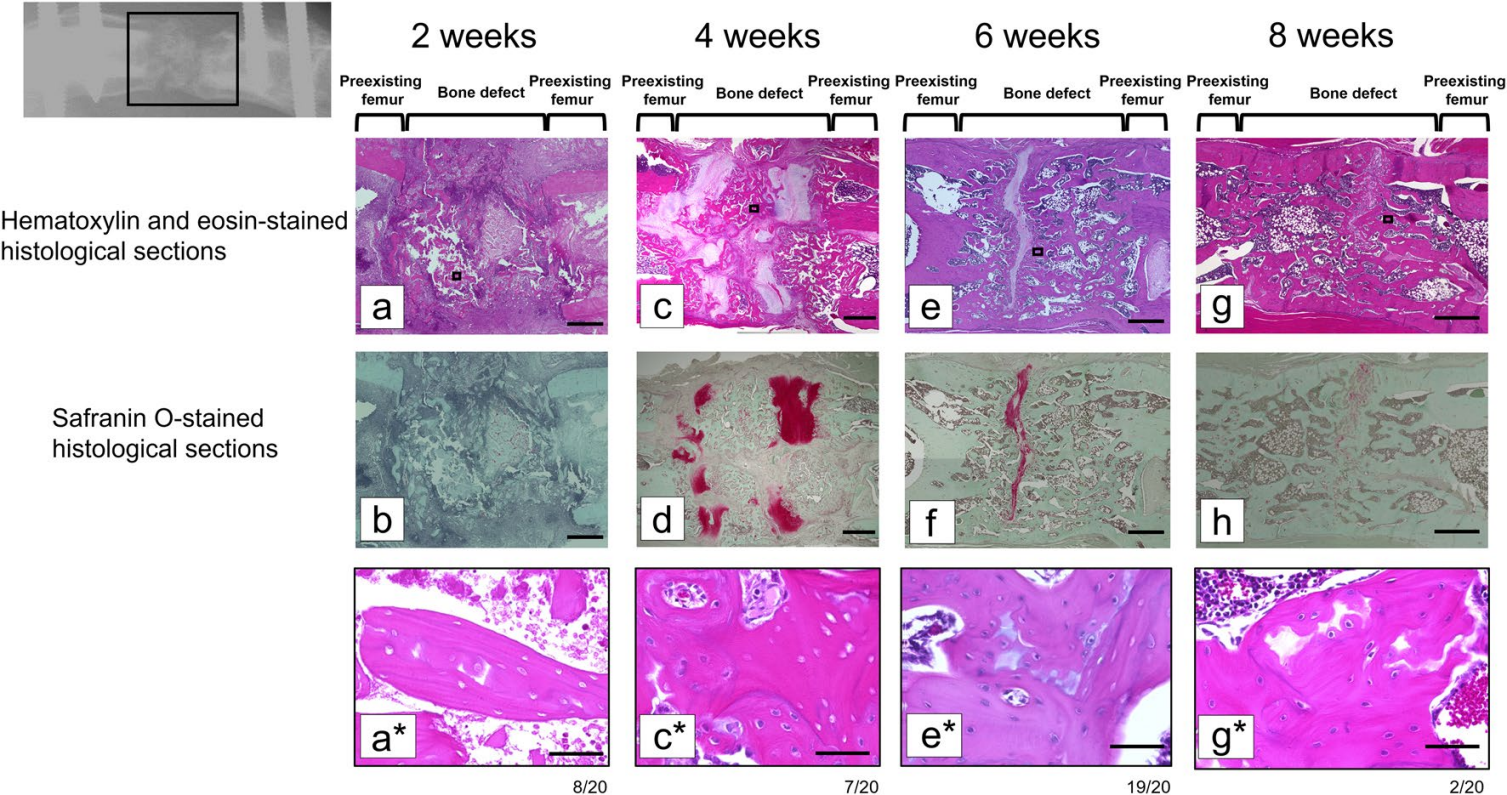
Histological analysis of the sagittal sections. (**a**–**h**) Magnified microphotographs of the sagittal sections (**a**,**c**,**e**,**g**) stained with hematoxylin and eosin and (**b**,**d**,**f**,**h**) Safranin O. Magnified images of the boxed regions in the upper panels (**a**,**c**,**e**,**g**). Scale bars: 1 mm (**a**–**h**) and 200 μm (a*,c*,e*,g*).

Sagittal sections stained with HE at 2 weeks post surgery showed that the grafted cancellous bone was located within the bone defect (Fig. 8a). Chondrocytes were absent in the Safranin O-stained sections (Fig. 8b). Two chondrocyte layers were evident in the bone defect, near the bone stumps, 4 weeks post surgery (Fig. 8c,d). We hypothesized that these layers converted the grafted cancellous bone into new bone in each bone stump. One chondrocyte layer positioned near the center of the bone defect at 6 weeks post surgery (Fig. 8e,f) may have been generated by a combination of the two chondrocyte layers observed 2 weeks earlier. By 8 weeks post surgery, the chondrocyte layer had nearly disappeared, and the new bone was connected on both sides (Fig. 8g,h). Under higher magnification, no nuclei were observed in bone cells at 2 weeks post surgery (Fig. 8a*), whereas bone cells without nuclei and live bone cells were mixed at 4 weeks post surgery (Fig. 8c*). At 6 and 8 weeks post surgery, all bone cells (autologous graft or new bone) in the defect were alive (Fig. 8e*,g*).

## Discussion

ACB is highly osteogenic, and it easily revascularizes and rapidly incorporates into host sites due to a large surface area covered with dormant and active osteoblasts^5^. Although ACB is an excellent space filler, it does not provide sufficient structural support^25–28^. Conversely, autologous cortical bone grafts provide immediate structural support^29,30^ but exhibit negligible osteoinductive potential^29,31^. Although rat models of autologous bone grafts have been previously described^32–34^, most of these grafts comprised the cortical bone. To the best of our knowledge, the model reported herein is the first to use only cancellous bone grafts in rats.

Regarding the safety of ilium collection, some studies have reported the use of bone grafts from the rat iliac bone^25,35^, but we could not confirm the safety details in these reports. Furthermore, the anatomy of the iliac bone as well as important parailiac structures such as the blood vessels and nerves surrounding the ilium has been previously reported^23^, but a safe osteotomy position for the ilium has been difficult to determine. Here, we minimized invasiveness during bone harvesting and increased the safety of our model by considering the morphology of the ilium and important parailiac structures.

The volume of cancellous bone that can be collected is vital for its application in multiple experiments. Here, the volume of the collected cancellous bone exceeded that observed in bone defects of several previous models. Moreover, the bone defects inflicted in models to improve bone formation can be classified as diaphysis, bone holes, and calvarial defects. Most rat models of bone holes^13,15,16,36^ and calvarial defects^14,34^ require some cancellous bone, and we believe that our model would fulfill such requirements. The length of the model diaphysis defect varies among studies^9,21,37–40^, ranging from 0.5^38^ to 8 mm^39^, whereas the diameter of the bone graft has not been reported. Artificial bone grafts with 3–4 mm diameter have been grafted^9,22,40^, from which the amount of ACB required for the femoral bone defect in rats has been calculated. We harvested a mean pure cancellous bone volume of 73.8 ± 5.5 mm^3^, which is sufficient to fill bone defects of diameter 4 mm and length ~ 5 mm (2 × 2 × π × 5 = 62.8 mm^3^) or diameter 3 mm and length ~ 8 mm (1.5 × 1.5 × π × 8 = 56.5 mm^3^). Hence, our model can be applied to nearly all known bone defect models.

In this study, as well as in models in which all rats presented nonunion in the absence of bone grafting^21,22^, autologous cancellous bone grafting led to good bone union, indicating that rat autologous cancellous bone grafting strongly promotes bone union. Regarding the role of each tissue in bone union, a previous study indicated that cells lining the endosteum and marrow stroma contribute to over half of the newly formed bones, and the contribution of osteocytes is approximately 10%^26^. In this study, all cells in the ACB graft were already dead at 2 weeks post surgery. However, a large surface area covered with active osteoblasts rendered ACB highly osteogenic, easily revascularized, and rapidly incorporated at host sites. Thus, 4 weeks post surgery, the cancellous bone with dead cells was readily covered by the new bone containing live cells, and this supports the findings of the abovementioned study. There are still several unclear aspects about the process of bone fusion by autologous cancellous bone grafting. We believe that one of the reasons is that it is difficult to pathologically examine the course of bone fusion over time in humans and large animals. This study confirmed, using a rat model, during the fusion of the ACB graft with the bone defect, two distinct chondrocyte layers that initially appeared in the bone defect near each bone stump and gradually became a single layer, extending toward the center of the defect. Although these chondrocyte layers may have been generated by micromotion during the external fixation used in this study, this process suggests the possibility of chondrocyte layer-mediated bone neoformation, protruding from the bone stumps toward the center of the defect, using the ACB graft as a scaffold. Nevertheless, this study was limited by the fact that osteoblasts or mesenchymal stem cells were not considered in the process of bone fusion by autologous cancellous bone grafting. Furthermore, we did not conduct histological, morphometric, and immunolabeling analyses to evaluate new bone formation. However, the key purpose of this study was to establish the first autologous cancellous bone graft rat model, and we plan to analyze the functions of these cells in new bone formation using comprehensive in vitro techniques in future studies. Another limitation was that we could not prove that this mechanism is similar to that occurring in humans.

Numerous strategies to improve bone healing have been reported, including the use of growth factors^41–44^, extracellular matrix peptides^45–48^, small regulators of bone mass^49–51^, and stem cells^52–55^; however, only a few therapies have been clinically translated. For the clinical translation of new strategies, it is essential to compare them with ACB grafts. To the best of our knowledge, this is the first attempt to develop a stable model of ACB fusion in rats by grafting ACB harvested from the iliac bone. This model will facilitate the exploration of neo-osteogenesis and technically complement the existing bone healing strategies and ACB grafts.

In conclusion, we established a stable rat model of ACB grafts by harvesting the iliac bone. This rat model can aid further investigation of ACB grafts and the future development of novel therapies for bone injury.

## Data Availability

All data generated or analyzed during this study are included in this published article.
